# THE JOURNAL OF APPLIED GERONTOLOGY: AN INTERNATIONAL PERSPECTIVE

**DOI:** 10.1093/geroni/igae098.0357

**Published:** 2024-12-31

**Authors:** Debra Dobbs

**Affiliations:** University of South Florida, Tampa, Florida, United States

## Abstract

The mission of applied gerontology is to bridge science and practice to benefit the health and well-being of older people, their families, their communities, and other contexts. Journal of Applied Gerontology features original investigations or meta-analyses/systematic reviews with significant clinical, policy, and/or practice implications. This presentation will provide insights from Journal of Applied Gerontology and its commitment to publish and disseminate scholarship with international application. Following an overview of the growing internationalization of peer-reviewed submissions to Journal of Applied Gerontology on a variety of topics and from a range of perspectives, the presentation will highlight opportunities to apply gerontological scholarship to aging contexts worldwide. Concluding comments will examine how outlets for dissemination and authors themselves can better position their work to enhance their influence on aging in an international context.

